# Valedictory Address of Dr. W. W. James

**Published:** 1879-03

**Authors:** 


### VALEDICTORY ADDRESS OF DR. W. W. JAMES.

*Honored Professors, Fellow Students, Ladies and Gentlemen:*

The Commencement exercises to-night close the twenty-second annual session of the Atlanta Medical College.

By the kindness of my colleagues I have been honored with the duty of addressing you, and in their behalf, as well as my own, I am here to say farewell.

I am confident that from this hour and this occasion we will sever some of the happiest associations of life, but around it will cluster the dewy blossoms of hope, and with it will ever linger some of the brightest memories, the most pleasant recollections of our lives. Everything pertaining to human life in its minutest details and in its grandest results, has its beginning and its end. The joys and sorrows, trials and triumphs of men and women of every age, in every condition, under every form of government, with every creed in religion, and in every type of civilization, have their end.

The practice of virtue ends in happiness—of vice, in misery; and the world's history is but the illustration of this vital truth.

Studious application ends in profound scholarship—negligent inattention in dull stupidity. Unholy ambition grasps at the bauble of fame, and seizes the emptiness of vanity, and the golden fruit upon which the hungry spirit would feed "turns to ashes upon the lips." Struggling genius combats the world's cold inattention to its sublime conception, which condemns its victim to a life of disappointment and often of beggary, and sets history ablaze with a flood of posthumous glory.

The conqueror drenches an empire in blood and wins the execration of mankind, while the patriot offers upon his country's shrine liberty's last libation, and emblazons his memory in historic immortality.

The end of sorrow is joyful, and the end of joy is sorrowful. This, ladies and gentlemen, is an occasion of mingled joy and sorrow. With high hopes and pulse-quickening anticipations, we are about to go forth upon the world's broad field of battle on our eventful missions.

But the beginning of our professional life is, for many of us, the end of our sojourn in your enterprising city.

We need not say our stay in your midst has been pleasant. We trust it has been profitable as well; and when the spirit of the future, linked to the chariot of the present, has borne us down the lane of life, may you, as well as we, look back to the sunny shores of by-gone years from the silvery strand of a shadowless clime, and cherish happy memories of us and our sojourn in your midst. Let me ask, too, that you will ever cherish our Alma Mater, and welcome those who are to come after us as kindly as you have greeted us.

To us the future is full of promise, and our floating barques are freighted with hope. This occasion fills our hearts with pleasant emotions, and inspires us with a feeling of our personal importance; for of course every young doctor feels that the physical destiny of mankind rests upon him! The very name carries with it no ordinary significance to a young gentleman just starting upon the practice of medicine. Yet, while these things produce in our minds a pleasureable sensation, the golden joy is not unmixed with an alloy of sorrow. Yea, there is a peculiar sorrow in ending pleasant relations, in severing happy associations, which in all probability are never to be renewed. This sorrow, however, is not peculiar to any age or condition. It melts the eye and thrills the heart of the student to behold his class separating to meet no more, just as it thrilled the heart and melted the eye of the great Washton, at the close of the Revolution, when he embraced for the last time those iron-hearted heroes who had stood by him in its perils and aided him in achieving its glorious results. Hence, it is with feelings oscillating between joy and sorrow, that I appear before you to-night.

To attempt to please your fancy by wandering with you through the flowery fields of rhetoric, would be to fail. To attempt to excite your mirth by a humerous speech would be futile.

To endeavor to delve in the vast repositories of human thought, for a new theme, with which to present to you, would be unpardonable presumption; I must, therefore, be content to bid you, dear citizens of Atlanta, a simple, sincere, unadorned farewell.

Fellow students, we are standing to-night upon the threshhold of an active life, and, as we peer anxiously into the mist of the coming years, it is but natural that our inexperienced minds should be filled with bright anticipations of success, honor and happiness, which we trust await us in the future. Notwithstanding the gray heads and furrowed brows of our teachers, which point like silent monitors, to the battles to be fought, the hardships to be endured, and the defeats to be borne, we glance eagerly at the arena of life, impatient to launch fearlessly amid the tide of contending spirits, in the fierce struggle against disease and oblivion.

The bright panorama of life is spread out before us. We have yet to play our part in the world's great drama, and it may not be amiss to consider the course which should distinguish our career.

Our profession is an absolute necessity. Our mission one of mercy. We are to be the good Samaritans who are to minister to suffering humanity, along the dusty wayside of life, and, upon the faithful performance of these duties, depend our future happiness and success.

It is, therefore, of the utmost importance that we should be thoroughly versed in the science of medicine.

This is by far the most enlightened period of the world's history, and he who wins distinction, and gains the confidence of his fellow men, must do so by a thorough knowledge of the profession he selects. I would not for a moment encourage an unholy ambition, which has for its sole object self-aggrandizement.

"For 'tis a glorious cheat,
Yet angels of light walk not so dazzingly
The sapphire walls of Heaven!
Earth's constellated thrones hath not such gems."

But like the beautiful and deceptive mirage upon the burning desert, it will allure the traveler from the highway of honesty and virtue, until exhausted and disheartened with the fruitless chase, he sinks beneath the waves of oblivion, with the maddening consciousness, that,

> "Doubly dying he shall go down
> To the vile earth from which he sprung,
> Unwept, unhonored and unsung."

Cæsar had such ambition, and the hand of his best friend struck home the fatal dagger, and his life blood paid the forfeit.

Napoleon, who bathed the fetlocks of his war-horse in the blood of his countrymen, flung the silken folds of his banner to the breeze, upon the cloud-capped summit of the Alps, faced unflinchingly the burning sands of the desert, and flashed back the sunbeams from his victorious falchion at the foot of the grand pyramids of Egypt—even Napoleon was a prey to this ambition. Upon the blood-stained field of Waterloo, the star of his glory went down in blood, and he died an exile upon the rock-bound shore of Helena.

Then my comrades, shun its baneful influence, as you would the blistering dew upon the deadly upas.

Ours is not a profession, in which bright laurels and brilliant blossoms of renown will bud and bloom in a day. We cannot

> "Dive to the bottom of the mighty deep,
> And drag up drowned honor by the locks,
> Nor soar into the region of the skies
> And pluck bright honor from the pale-faced moon."

Still with a thorough knowledge of our profession, a heart to pity, and a hand to help suffering humanity, we may leave an impress upon the minds of the people, that shall never be obliterated, until the withered hand of Time shall fall powerless to his side, and nations revel in eternal youth.

Fellow Students, we stand *only* upon the threshold of our professional life. Our medical education is but just commenced, and to fit ourselves for the duties and responsibilities of the future, will require patient application, untiring energy, and thorough mental culture. Let us not be deluded with the idea that simply because we have our diplomas we may sit inertly in our offices and business will come to us.

*Time was* when a man could boastfully pretend to a knowledge which he did not possess, and could abuse the confidence of an ignorant public, but that time has passed. Ignorance is fast giving place to intelligence, and the man who now succeeds must do so upon the basis of merit.

"Let us, then, be up and doing,
With a heart for every fate,
Still achieving, still pursuing,
Learn to labor and to wait."

Ours is a noble profession, and has numbered in its list of votaries some of the proudest names in history. He who practices medicine upon principles of truth, honor and fidelity, may be absolutely certain of a useful life and an honorable reputation that will live

"To win the wreath of fame,
And write on memory's scroll a deathless name."

Yet we cannot hope to find the "gems of the beautiful" scattered all along our pathway, nor expect to shun the thorns while we gather the sweet-scented rose.

We will often meet with disappointment, and our most earnest endeavors be sometimes crowned only with defeat; but by an unswerving integrity of principle a thorough development and culture of our minds and the exemplification of a noble manhood, we may leave such a record that not only our friends may admire, but even the world may say,

"I've scanned the actions of his daily life
With all the industrious malice of a foe,
And nothing meets mine eyes but deeds of honor."

And now, honored Professors, this hour which dissolves the relation existing between us as teachers and students—a relation which has associated us in labor and allied us in friendship—a relation that has imposed upon you the high and important duty of fitting us for the responsibilities of life—this hour, which severs such relations, brings to me the happy privilege of returning to you our earnest thanks for your untiring efforts in our behalf.

As each sacred tendril is broken from the heart, it stamps with its flowing blood-drops the tablets of memory; so when the chain is rent and our barques float out on life's ocean, and the high waves from time's deep sea have swept between us, we will ever hold in grateful remembrance the kindness which you have ever shown us.

The fidelity which you, our respected professors, have displayed in the execution of this sacred trust, evinces your appreciation of its magnitude.

The principles of science which you have inculcated, will, we trust, be the basis upon which, by industry and application, we may rear a noble superstructure, but of which you will still be the architects. The discipline, through which we have passed, will aid us in future, as we ramble through the vast and unexplored fields of science.

We do not feel discouraged at the reflection that there is still so much for us to learn, but rather stimulated to renewed exertions, assured that patient, persistent and energetic effort will enable us to reach the goal of our aspirations, and reflect credit and honor upon our Alma Mater.

Through these long years, while we, remembering "there is room at the top," strive to ascend the hill of life and of fame, may the uncertain future steal softly from out the gloom, and so gently unroll time's printed page, that his hand may fling bright hours around your way, and his latest footstep bring only blessings for you; and may we never rest low in oblivions dell, until that time, when far from earth's joys and cares, we hope to meet—nor ever say farewell.

And we, my comrades, who have in the past, shared such pleasant associations, formed such warm attachments, and enjoyed such genuine friendship, will, as we disperse and go forth upon the world's broad field of battle, bear with us joyous recollections, and enshrine within our hearts happy memories, to which we can revert in after years, when we perchance may be weary with the bivouac of life.

These pleasant reminiscences will shed their light upon the deepest gloom of the untried future; and in the darkest hours, when our hearts are faint, as the captive Israelite, upon the bank of the Euphrates, turns his anxious gaze to the vine-clad hills of native Palestine; so we, my fellow students, borne in the tender arms of memory, may return to these consecrated scenes, and bathe our spirits in the joys forever fled.

If we never more meet within the halls of our beloved Alma Mater, let us strive to honor our teaching, and be true to the noble principles taught us by these esteemed preceptors.

Let us remember, always, that hearts had ne'er been supernal, without a world to brave; and when life's leaves shall wither, may we have gathered many gems of virtue; and when the bloom of life's coronal has faded, may we weave living circles around immortality.

Till then, my class-mates, though the leaflets of memory, hang dripping with tears, while we speak the word—yet we, too, must say *farewell!*

